# Biocompatible Nanocomposite Implant with Silver Nanoparticles for Otology—In Vivo Evaluation

**DOI:** 10.3390/nano8100764

**Published:** 2018-09-27

**Authors:** Magdalena Ziąbka, Elżbieta Menaszek, Jacek Tarasiuk, Sebastian Wroński

**Affiliations:** 1 Department of Ceramics and Refractories, Faculty of Materials Science and Ceramics, AGH University of Science and Technology, 30-059 Krakow, Poland; 2 Department of Cytobiology, Collegium Medicum, Faculty of Pharmacy, UJ Jagiellonian University, 30-001 Krakow, Poland; elzbieta.menaszek@uj.edu.pl; 3 Department of Condensed Matter Physics, Faculty of Physics and Applied Computer Science, AGH University of Science and Technology, 30-059 Krakow, Poland; tarasiuk@agh.edu.pl (J.T.); wronski@fis.agh.edu.pl (S.W.)

**Keywords:** nanocomposites, medical devices, middle ear prosthesis, silver nanoparticles, biocompatibility, thermoplastic polymer

## Abstract

The aim of this work was to investigate of biocompatibility of polymeric implants modified with silver nanoparticles (AgNPs). Middle ear prostheses (otoimplants) made of the (poly)acrylonitrile butadiene styrene (ABS) and ABS modified with silver nanoparticles were prepared through extrusion and injection moulding process. The obtained prostheses were characterized by SEM-EDX, micro-CT and mechanical tests, confirming their proper shape, good AgNPs homogenization and mechanical parameters stability. The biocompatibility of the implants was evaluated in vivo on rats, after 4, 12, 24 and 48 weeks of implantation. The tissue-healing process and cytotoxicity of the implants were evaluated on the basis of microscopic observations of the materials morphology after histochemical staining with cytochrome c oxidase (OCC) and acid phosphatase (AP), as well as via micro-tomography (ex vivo). The in vivo studies confirmed biocompatibility of the implants in the surrounding tissue environment. Both the pure ABS and nanosilver-modified ABS implants exhibited a distinct decrease in the area of granulation tissue which was replaced with the regenerating muscle tissue. Moreover, a slightly smaller area of granulation tissue was observed in the surroundings of the silver-doped prosthesis than in the case of pure ABS prosthesis. The kinetics of silver ions releasing from implants was investigated by ICP-MS spectrometry. The measurement confirmed that concentration of the silver ions increased within the implant’s immersion period. Our results showed that middle ear implant with the nanoscale modification is biocompatible and might be used in ossicular reconstruction.

## 1. Introduction

The need to replace or reconstruct ossicles has led to the development of surgical techniques enabling innovative prostheses implantation. New structural and material possibilities have improved the design and preparation of prostheses so as to make them vary in size, shape and the applied material. Nowadays, it is common knowledge that a well-designed material may result in a more advantageous postoperative response. Moreover, proper modifications of the chemical composition change the parameters and functions of mechanical prostheses.

The ossicular chain reconstruction may be carried out with either partial ossicular replacement prosthesis (PORP) or total ossicular replacement prosthesis (TORP). Unfortunately, many ossicular chain reconstructions—using either PORPs or TORPs—still fail. There are various factors determining the success of the operations, such as the proper length of the prosthesis, stability of implantation, recurring illnesses, risk of inflammation, and reaming out the ear to provide passage of air [1,2]. Another important factor is the presence of either anatomic incus or stapes that facilitate the stability of the prosthesis fixation. Despite the possible difficulties and complications, both partial [3,4,5] and total prostheses [6] are effective in ossicular chain reconstructions.

The research conducted for the last 40 years has thoroughly described the requirements set for materials used in laryngological surgeries. The most important issue is the optimal quality of sound transmission that is influenced by various biological, acoustic and mechanical factors. As far as the biomechanical functions of the device are concerned, the mechanical properties of the implant material are key factors. Still, it is possible to tailor the material to specific needs depending on the implantation site, the size of the implant and the manner of manufacturing. The materials for middle ear prostheses do not face precise strength requirements. Yet it is common knowledge that the implant material should have such mechanical properties so as to best resemble the tissue it is supposed to substitute. It is a challenge to design a perfect material, considering the complex structure and the chain of auditory ossicles, as well as the wide spectrum of Young’s modulus for particular elements (e.g., ligaments, muscles, joint and bones) which ranges from 0.049 MPa (for ligaments) to 14 GPa (for bones) [7]. The transmission of high-frequency sound depends on such parameters as the surface of the prosthesis, its stiffness (rigidity), Young modulus, Kirchhoff modulus, friction and the implant’s density and weight (mass) [8,9,10]. Although the lightness of the structure is connected with the type and size of the implant, the essential factor is the material of the prosthesis. In order to provide the best quality of high-frequency transmission, the implants ought to be as light as possible—the higher specific gravity of the implant, the lower its high-frequency sensitivity [11]. Apart from the sound transmission of the middle ear implant, the biological functions of the material are a key requirement in medical applications.

The mechanical properties of the material dedicated for ear implants are also clearly defined. The biomaterial is supposed to sustain its shape, constant measurements, proper elasticity and rigidness for the longest possible period of time. The material should be also resistant to changing loads and prove its high resilience in fatigue tests. Additionally, middle ear prostheses must be capable of making micro movements between the eardrum and middle-ear chamber.

Polymeric/(poly)acrylonitrile butadiene styrene (ABS) materials play a significant role in bone surgery and laryngology [12]. There were a few reasons for selecting high ABS as a material for prototype implants. First of all, it is very convenient to obtain complex shapes by means of injection moulding. ABS polymers can be modified with silver nanoparticles, obtaining the following advantages: bactericidal efficacy against *Staphylococcus aureus* and *Escherichia coli*, slight but visible cytotoxicity against fibroblasts (ensuring better implant-bone fixation without scarring), no cytotoxic activity against osteoblasts, advantageous mechanical properties, fatigue stability, high homogenization of nanosilver in the polymer matrix and a high level of silver ions released into the environment [13]. All the previously mentioned factors proved Ag-modified ABS to be a very promising material for a prototype of the middle ear implant.

The potential for the use of nanoparticles in surgery is huge. Antibacterial properties of silver nanoparticles are used in urology, implantology and dentistry, as well as to treat burns or other chronic wounds [14,15]. For instance, catheters can be coated with silver nanoparticles to endow them with antibacterial properties and prevent surface biofilm formation [16]. In the surgery of ossicular replacement prosthesis, none of the implants possess bactericidal properties. Nowadays, the range of commercially available materials used for bone reconstruction is impressive. On the market, the most popular group of materials used for such prostheses are metals (titanium), ceramic (hydroxyapatite), polymers (PTFE-teflon) and some composites (HAPEX) [17]. According to the literature reports, the titanium prostheses display better biostability and biocompatibility in comparison to allogenic grafts. The titanium implants sustain proper stiffness and they are efficient in sound transmission and lightweight, which is a vital factor in the postoperative assessment [18]. The ossicular chain reconstructions are often performed with hydroxyapatite prostheses, as an alternative to auto- and homografts. They are popular mainly due to the biological aspect. It is one of the main components of bones and teeth and it forms a stable implant/tissue bonding without the fibrous layer around the prosthesis. Hydroxyapatite also stimulates the cell proliferation and is highly biocompatible [19]. The negative feature, however, is the formation of a big mass in the relatively small middle ear cavity [20]. Teflon is used to obtain partial ossicular replacement prosthesis, ventilation tubes and drains, incus and stapes prostheses (piston type) usually with a platinum wire. Due to its hydrophobicity and low surface energy teflon is especially popular for the stapes prostheses [21]. One of the most popular materials for the ossicle chain reconstruction is HAPEX. It is composed of 40% synthetic hydroxyapatite (HAp) and 60% high-density polyethylene (HDPE). In the stress tests, HAPEX has proven to be a stable implant/bone bonding. Fibrous tissue formation was observed on the implant surface and the implant/bone border, in some cases a thin epithelium layer outside was also observed [22].

In our case, the whole prosthesis is made of a thermoplastic polymer (ABS), which makes it lightweight. It is also possible to adjust the implant’s length. The round shape of the head plate minimizes the risk of tympanic membrane damage. The openwork construction of prosthesis (antenna) allows its easy placement in the middle ear and creates an opportunity to manually form a desired shape, according to the particular ossicular chain damages. Moreover, the mechanical properties, such as Young’s modulus, are similar to the bone. Additionally, the cheap manufacturing method makes the product competitive in the scope of general costs of treatment. The novelty is also the antibacterial function of the plastic prosthesis. This medical device is similar to the titanium prosthesis in shape but, up to now, it has never been manufactured by injection moulding and extrusion. Therefore, antimicrobial polymers are highly demanded as a strategy to avoid otitis media infections.

The perfect prosthesis material should be biocompatible with the surrounding tissues, it cannot result in acute immunological, toxic, or allergic reactions [23]. Moreover, it should not display mutagenic or carcinogenic effects. From the biological point of view, especially in the case of chronic middle ear infection, the material’s stability in the environment is an essential quality too. The material should neither degrade nor facilitate the further inflammation process [24,25]. It should be endowed with proper wettability value to facilitate the epithelial cells proliferation, which guarantees the successful adaptation of the implant.

According to the correct sequence of biological research, the implant material should first undergo the in vitro cytotoxicity testing procedures. Then it should be tested on animals to describe an interaction with the soft tissue and, preferably, also in the environment corresponding to the one of the middle ear [26].

In this work, the medical devices made of nanocomposite with antibacterial silver nanoparticles have been described as valid tools for otolaryngology. The biocompatibility of these devices has been tested in vivo. Our results showed that this micro-device with the nanoscale modification is biocompatible and very promising as a novel middle ear prosthesis.

## 2. Materials and Methods

### 2.1. Material Manufacturing

**Figure 1:**
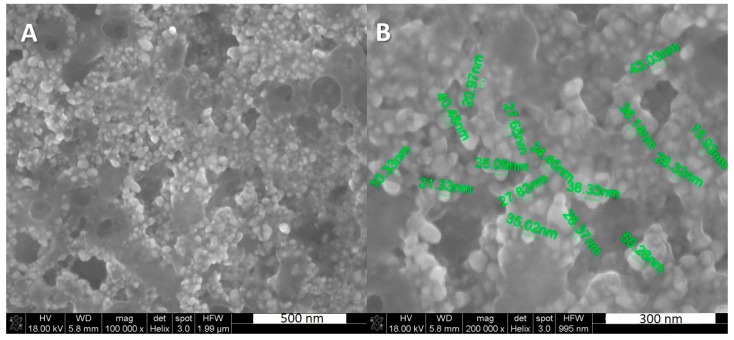
SEM images showing the silver nanoparticles, (**A**) silver nanoparticles (AgNPs), scale 500 nm, (**B**) AgNPs with visible diameter, scale 300 nm.

The otoimplants were manufactured by means of extrusion and injection moulding method, using the Multiplas machine (Multiplas Enginery Co., LTD, Taiwan) fitted with a special steel moulding form. The two types of the implants were injected: (poly)acrylonitrile butadiene styrene (ABS) and ABS with the addition of 0.1 wt. % silver nanoparticles (AgNPs). The silver was developed at the Intercollegiate Faculty of Biotechnology, University of Gdansk and Medical University of Gdansk manufacture according to Banasiuk et al. 2016 [27]. The size and shape of nanoparticles were estimated via SEM and TEM [27]—they were characterized as spherical and measuring below 50 nm in diameter (Figure 1). The AgNPs were agglomerated as an aqueous environment evaporated during the procedure of sample preparation for SEM and nanoparticles started to aggregate.

The procedure of obtaining the prostheses according to Ziąbka et al. 2017 [28] consisted of a few steps. First, the granulate was prepared and dried in the laboratory dryer at 80 °C for 6 h. Next, the nanosilver particles were incorporated and homogenized with polymer granules in the plasticizing chamber using a 0.8 m-long screw. Subsequently, the material was injected into the steel moulding form, cooled and extracted. The injection parameters were selected and adapted for the process according to the characteristic data sheet of the polymer manufacturer (injection temperature in three zones—240 °C, injection pressure—80 kg cm^−2^, flow—70%).

**Figure 2:**
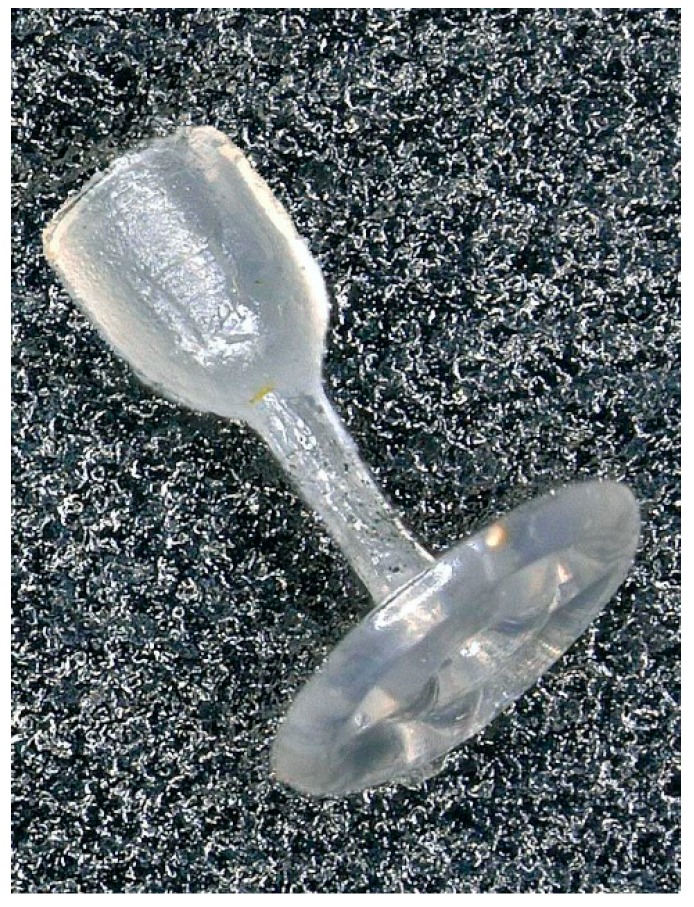
The middle ear implant—otoimplant.

The shape of our otoimplant (Figure 2) does not vary significantly from the other prostheses, as it has to replace ossicular chain bones and easily fit in the middle ear. However, we have enhanced some of its parts to simplify the surgical procedure. The shape we developed is surgically handy, ensuring the precise implantation in the middle ear.

The prosthesis consists of the three elements: A “cup” which is placed on the head of the stapes, a “piston”—joining the cup and an “antenna”—the implant base which bends on the tympanic membrane. The openwork construction of the antenna determines the implant weight and expedites the implantation.

### 2.2. Material Evaluation

#### Scanning Electron Microscopy

The SEM-Quanta FEG-250 scanning electron microscope (FEI, Eindhoven, The Netherlands) was used to perform a detailed examination of the otoimplants microstructure. The measurements and observations were conducted in high vacuum conditions, with a back scattered electron detector (BSE), with the accelerated voltage of 10–18 kV. The microstructure observations were conducted on two kinds of the implants—one was made of pure ABS and the other of ABS modified with silver nanoparticles. All the samples were coated with a carbon layer.

Additionally, the microstructure of these implants was investigated using the Nova Nano SEM 200 scanning electron microscope (FEI, Eindhoven, The Netherlands) coupled with a Genesis XMX-ray microanalysis system (EDAX, Tilburg, The Netherlands). The measurements and observations were conducted in low vacuum conditions with a secondary electron detector (SE), the accelerated voltage was 10–18 kV. The samples were coated with a carbon layer.

### 2.3. Implantation Procedure

The procedure of implantation was performed at the Animal Facility of the Faculty of Pharmacy CM UJ Krakow (the consent no 251/2015 issued by the 1st Local Ethical Committee on Animal Testing in Krakow). The experiment was performed according to the PN ISO 10993-6 guidelines [29]. The male adult Wistar rat (*Rattus norvegicus*) was chosen as a research model. The animals were kept in standard conditions at the stable temperature of about 20 °C and the 12:12 h light cycle.

**Figure 3:**
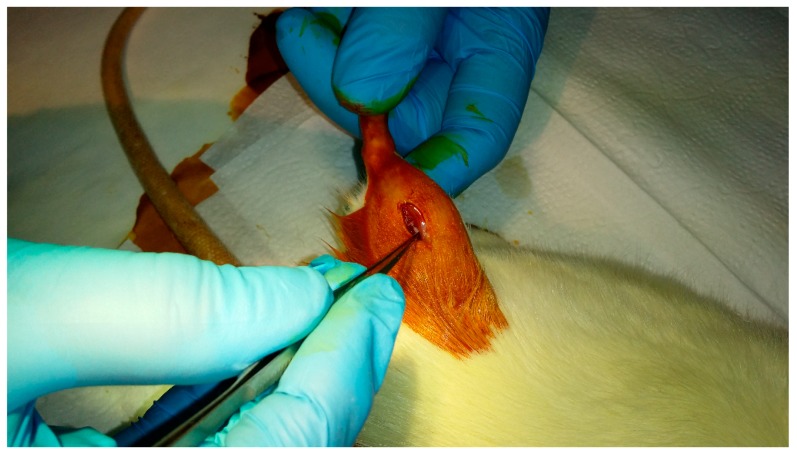
Implantation procedure of middle ear prosthesis into the rats’ gluteus muscle.

The middle ear prostheses made of pure ABS and silver-doped ABS were sterilized at a low temperature gas plasma (the Sterrad 120 apparatus) using hydrogen peroxide vapour in the double-cycle (2 × 45 min) and implanted into the rats’ gluteus muscles. The animals were divided into four groups for 30, 90, 180 and 360-day cycles, 5 rats in each batch. Prior to the surgery, the animals were sedated with the intraperitoneal injection (Ketamine + Xylazine: 100 mg/kg + 10 mg/kg animal body weight) and the implantation area was shaved and disinfected with iodine. Next, a small incision was made in the skin and the underlying muscle to create a small pouch (3–4 mm deep) where the implant was inserted. Then the double stitching was applied (degradable PDS II Johnson & Johnson Intl) to complete the surgery. The implantation procedure is presented in Figure 3.

### 2.4. In Vivo Examination

After a set period of time (30, 90, 180 or 360 days) the rats were decapitated, then the tissue samples were extracted and prepared (frozen in liquid nitrogen, cut into 9 μm-thick slices with a cryostat microtome—Shandon, Thermo Sci., GB) for histoenzymic and microstructural assessment. Additionally, the lymph glands adjacent to the implant site were extracted for further examinations. The histoenzymic reactions such as cytochrome c oxidase (OCC) and acid phosphatase (AP) were performed to identify the response to a foreign body and to assess the healing process. Slides performed for the AP activity were also stained with Mayer’s hematoxylin for better visualization of tissue structure. The observations were conducted using an optical microscope (Olympus BH2, Tokyo, Japan, objective 4–20×) and images were taken with a digital camera. In each series, the tissue samples containing the pure ABS implant and the ABS/AgNPs implant were extracted and immersed into formalin for the further micro-CT study. The samples of blood were harvested for CRP (C Reactive Protein) examination as well.

### 2.5. C Reactive Protein Measurement

Blood was collected directly from the heart of 5 rats decapitated after 30 days of implantation. The C reactive protein (CRP) concentration in blood serum was measured by an immunoturbidimetric method using the Cobas 8000 machine (Roche Hitachi, Mannheim, Germany).

### 2.6. Micro-CT Observation

The rats’ muscle tissues containing the implants of the two kinds (pure ABS and ABS/AgNPs) were harvested and fixed in 4% buffered formalin to perform the Micro-CT observations. The tests were performed 30, 90, 180 and 360 days after the implantation. All the samples were scanned in wet conditions at room temperature using a Nanotom 180N device (GE Sensing & Inspection Technologies Phoenix X-ray Gmbh, Grasbrunn, Germany). The micro-CT system provided a unique spatial and contrast resolution due to the installed ultra-high performance nanofocus X-ray tube (180 kV/57 W) and the tungsten target with a diamond window. The working parameters of the X-ray tube were I = 200 µA and V = 70 kV. The magnification was set to 6.7, which corresponds to 7.5 µm resolution. Each projection was averaged from five expositions taking 500 ms for each. The total number of projections was 1800. The reconstruction of the scanned implants was performed with the aid of proprietary GE software datos X ver. 2.1.0 using the Feldkamp algorithm for cone beam X-ray CT. The post-reconstruction data treatments, such as denoising, thresholding and visualization, were run in VG Studio Max.

### 2.7. ICP-MS

The pure polymer otoimplants and silver-doped otoimplants were incubated at 37 °C in 50 mL of UHQ water for one year. The ions release observations were also carried out on bigger samples (10 mm in diameter) due to the low concentration. The silver ions concentration was also examined in the blood harvested from the rats’ hearts one month after the implantation. The in vitro release of silver ions was studied by means of inductively coupled plasma mass spectrometry (ICP-MS), using the ICP-MS Perkin-Elmer Plasma 6100 spectrometer. Prior to performing ICP-MS analysis, so as to prohibit the silver ions (Ag^+^) reduction into metallic silver, the filtered samples were acidified with nitric acid, up to the final concentration of 0.1 mol/L. The silver concentration values of the investigated samples were determined using ICP-MS at *m*/*z* 107, applying the external standard calibration procedure.

### 2.8. Mechanical Tests

The mechanical parameters were established during the uniaxial stretching, using the universal testing machine Inspekt Table Blue 5 kN (Hegewald & Peschke GmbH, Nossen, Germany) and the intelligent testing software LabMaster. The tested samples were shaped as paddles made of ABS polymer and the silver-modified composite. Their measurements are compliant with PN-EN ISO 527-1 norm [30]. In order to perform the tensile strength test, the paddles were placed in the grips of the testing machine and the tensile force F was applied. The measuring speed of the upper grip of the machine was 50 mm/min and the measuring length of the paddles was 40 mm. The measuring accuracy of elongation was 0.01 mm, and of the force—0.5 N with the nominal range of the cylinder—5000 N. The obtained force-deformation graph made it possible to establish such parameters as Young’s modulus E, tensile strength σ and elongation at the maximum εFmax force.

### 2.9. Statistical Analysis

The results were analyzed using the one-way analysis of variance (ANOVA) with Duncan post hoc tests, performed with Statistica 13.1 (Dell Inc., Round Rock, TX, USA) software. The results were considered statistically significant when *p* < 0.05.

## 3. Results and Discussion

### 3.1. Microstructure Observation of Middle Ear Prosthesis

**Figure 4:**
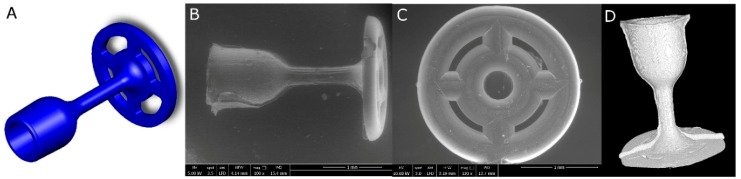
Middle ear prosthesis, (**A**) 3D model, (**B**,**C**) SEM images showing the implant from different sides, (**D**) prosthesis 3D reconstruction after micro-CT scanning.

The observations of the implants’ microstructure using the SEM method and micro-CT reconstruction confirmed the proper prosthesis shape obtained in the injection moulding process (Figure 4B–D). All the prostheses elements (the cup, the piston and the antenna) were of a homogeneous and consistent structure in comparison to the 3D model prepared in the Solidworks (Figure 4A).

An innovative solution is the openwork construction of the antenna that allows easier implantation and determines the weight of the implant. Initially, the complex mould shape and the small size of the medical device caused many difficulties. However, the well-thought-out injection moulding conditions and the efficient silver nanoparticles dispersion in the polymer matrix resulted in developing laryngological implants. Moreover, the implants were obtained in accordance with the design assumptions and the connection of the individual elements was proper. It is common knowledge that good dispersion and strong interfacial interactions between the nanoparticles and the polymer matrix are critical to engineering a strong composite. Therefore, it is a challenge to obtain the sufficient dispersion of hydrophobic fillers in a polymer matrix. Although various surfactants are widely used as they enhance dispersion, their cytotoxicity limits the biomedical applications [31]. Therefore, in our research, we decided against using any surfactants or toxic chemicals.

### 3.2. Ex Vivo Investigations of Tissue-Implants Samples

**Figure 5:**
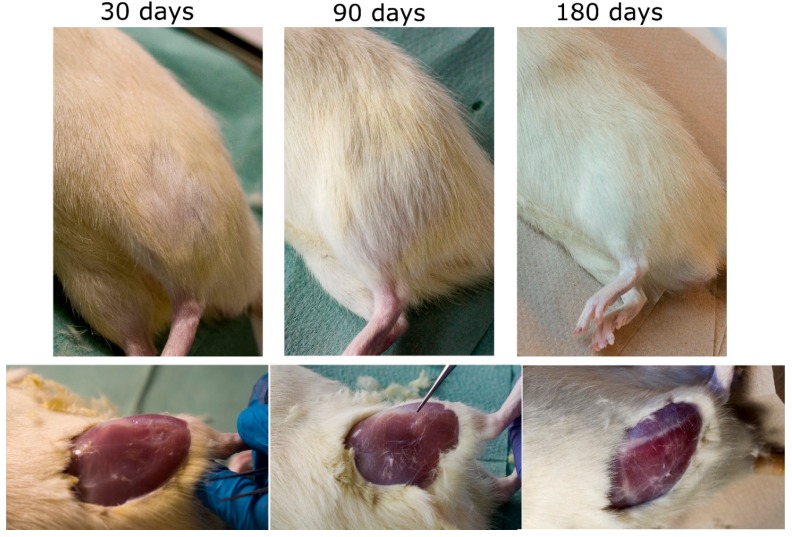
Implantation site after 30 days (**left**), 90 days (**center**) and 180 days for otoimplant/AgNPs.

All the rats not only survived the procedure but the in vivo tests did not reveal any postoperative complications or external symptoms of inflammation, e.g., reddening and irritation in both types of implants. After the 30-day postoperative period, the fur was growing back on the implantation site, whereas after 3 months the spot was fully covered in hair (Figure 5). There were no signs of scars or stitching left. Having removed the skin, the tissue with the area of surgery was visible. After 30 days a so-called “lens”—the place where the tissue collapsed—was easy to identify. From 90 days of implantation, the tissue looked absolutely ordinary. No macroscopic differences were to be observed between the two types of implants.

The internal organs of the rats were examined too. No changes in morphology were noted in kidneys, spleen, liver or intestines. The tissue samples were extracted for further microscopic and histochemical observations.

### 3.3. Histochemical Analysis

**Figure 6:**
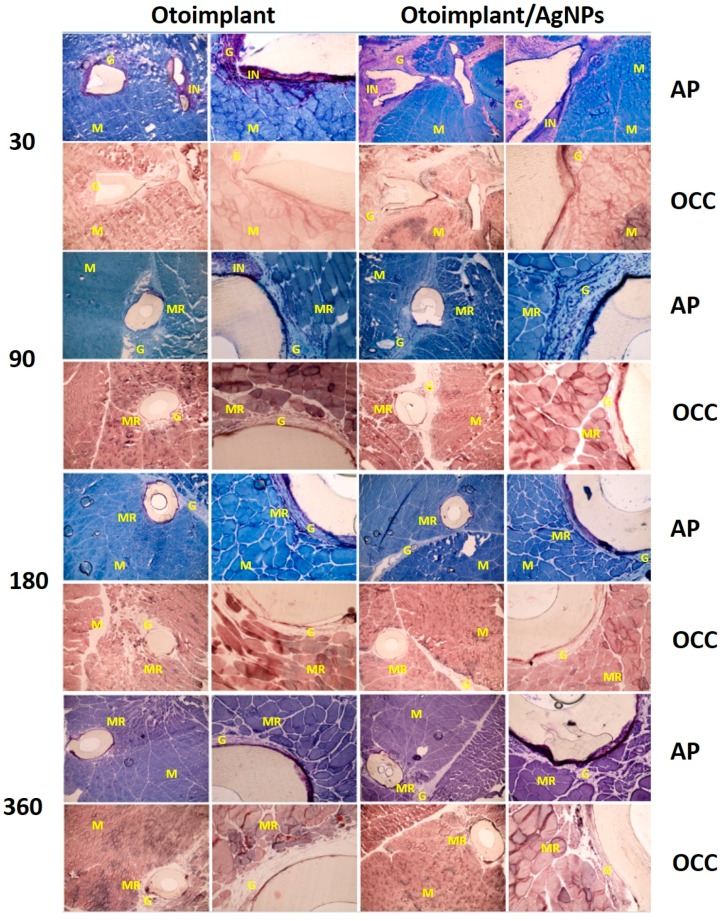
Cross-sections of tissues with otoimplant (on the **left**) and otoimplant/AgNPs (on the **right**) after 30, 90, 180 and 360 days of implantation in the rats’ muscles, acid phosphatase (AP) and cytochrome c oxidase (OCC) staining, objective 4, 20×. NOTE: The following symbols are used to describe the tissue cross-sections: M—muscle tissue, G—granulation tissue, IN—inflammatory infiltration, MR—regenerating muscle.

The in vivo histochemical tests (OCC and AP) conducted on the animals answered the questions concerning the cytotoxicity of the implanted material. OCC is an enzyme with high sensitivity to xenobiotics and its normal activity shows that the presence of an implant or ions released from its surface does not inhibit the metabolism of surrounding tissues. AP activity tests were carried out to demonstrate the intensity of inflammation caused by the presence of the implant in the tissue. The surgical insertion of the material resulted in an immunological response—first, it was the reaction to the surgery itself, then to the implanted material. In our study, moderate inflammation was observed around both types of implants (Figure 6). The presence of immune cells (granulocytes, macrophages and lymphocytes) involved in healing processes and the rejection of a foreign body was observed. As a result, the granulation phenomenon took place. The granulation tissue was being gradually replaced by the regenerating muscles and—subsequently—with the mature fully-developed muscles. The scar tissue formed only in the place of surgical incisions.

In some cases, there was an acute inflammatory infiltration. It is worth noting that both kinds of implants (ABS and ABS/AgNPs) led to similar immunological reactions, i.e., a lownumber of mast cells and eosinophiles. The observed inflammation resulted from the tissue damage and was the natural response to a foreign body. The local inflammation was observed around the prosthesis in the tissue samples obtained a month after the surgery and sectioned for histochemical analysis (Figure 6).

The C Reactive Protein tests (CRP) did not show any local inflammatory findings, as the AgNPs prostheses were too small to cause a negative response in blood. No inflammation or toxic effect was observed, which means that the concentration level of bioactive particles was safe. The mean value of CRP acute protein concentration was below the lower reference value in all the five investigated cases. CRP values in the investigated group were determined to be below 1 (reference value < 5.0). These values proved the lack of inflammation signals in the tested blood after 30 days of the implantation, which confirmed that the low concentration of silver ions could not affect the metabolic parameters of the blood. Therefore, no more CRP tests were conducted for longer experimental series. The elevated CRP, beyond being a biomarker of inflammation, may reflect the molecular disease mechanisms. For example, the CRP production by hepatocytes—the main source of the acute-phase reactant—appears to be regulated primarily by the proinflammatory cytokines interleukin (IL)-6 and IL-1. Furthermore, CRP itself has the ability to activate the complement system and enhance phagocytosis via opsonization [32].

**Figure 7:**
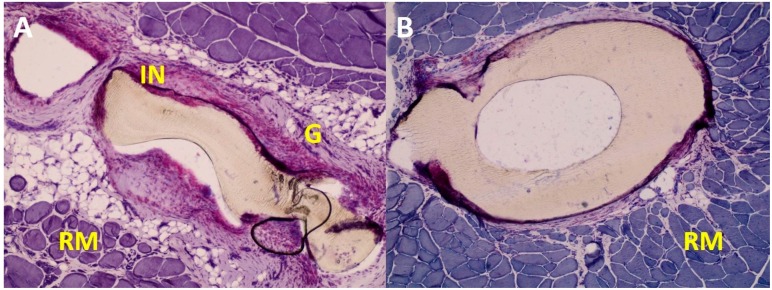
Exemplary images of the tissue around otoimplant after 360 days after the implantation. AP staining, magnification 10×. The images show the differences in the reconstruction of tissues around the implant parts: the antenna (**A**) and the cup (**B**).

During the studies, it became evident that the tissue response to the implant is largely dependent on the prosthesis shape. The acute inflammatory infiltration was observed more frequently in the antenna part than in the cup part of the implant. It seemed that the elaborate antenna design constricted the flow of juvenile tissues into its interior, thus the proper tissue growth was hindered in the inner part of the implant (Figure 7).

During the first month, the inflammation occurred mainly due to the surgical procedures and the implant presence. The implant instability—as it could still move inside the pouch in the rat muscle—might have been another reason for the inflammation. During the recovery the muscles were active and their constrictions would push the prosthesis outwards. The irritation of the muscle tissue was also caused by the obvious firmness and rigidness of the foreign body as well as the diversified shape of the implant. Still, despite the complex and gossamer design of the antenna, after 90 postoperative days, the granulation cells emerged in the spaces between the antenna elements.

**Figure 8:**
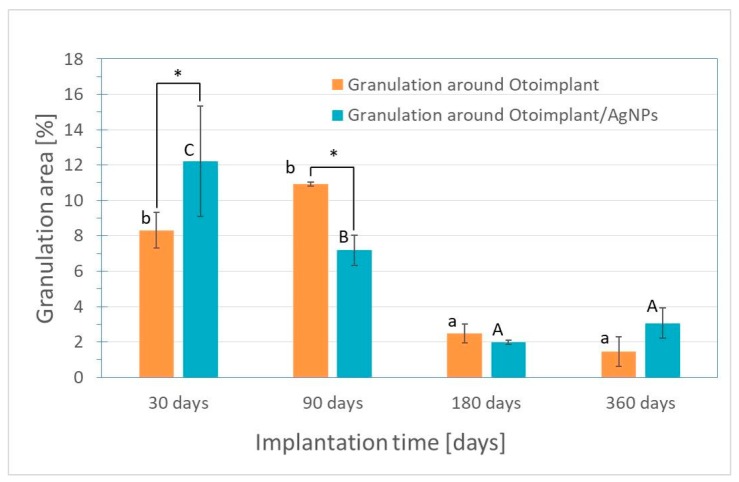
Granulation area for otoimplant and otoimplant/AgNPs measured after 30, 90,180 and 360 days of the implantation. Statistically significant differences (*p* < 0.05) between otoimplant and otoimplant/AgNPs after specific implantation time are indicated by *; between different implantation times for otoimplant and otoimplant/AgNPs—by a–b and A–C, respectively.

The microscopic observations led to the conclusion that the inflammatory reaction was weaker for the samples extracted after the 3-month implantation than for samples after 30 days of implantation. There was a significant decrease in the granulation tissue area for otoimplant/AgNPs prosthesis. (Figure 8). The regenerating muscle tissue was present at the implantation site and the first visible differences were noted between the two implant types after three months. The continuing inflammation was connected with the surgery and the presence of the foreign body, regardless of the material properties. The tissues far from the implantation site were a properly ordered mosaic, typical for skeletal muscles.

The implants were surrounded by the granulation tissuebut in some places were in close contact with the regenerating muscle tissue. However, in the case of the AgNPs-modified otoimplant the granulation tissue area was visibly smaller and it was getting replaced by the regenerating muscle tissue, thus suggesting that the presence of silver nanoparticles facilitated the healing process.

The inflammation diminished significantly around the implant after 180 days of the implantation. In the case of both the otoimplant and the silver-doped otoimplant, the granulation tissue areas diminished. However, the area of granulation tissue seemed to be slightly smaller for the Ag-modified prosthesis.

After 360 days of the implantation, the granulation remained on a constant level in comparison to the measurement after 180 days. To prove that silver nanoparticles may accelerate healing processes the area of granulation was measured in the Image J program and presented in a diagram as the results of the average values (Figure 8).

The in vitro tests revealed more numerous population of living cells and thus—lower cytotoxicity of the Ag-modified implants. The results indicated that the small number of nanosilver particles released into the surrounding tissue not only had a bactericidal effect but also stimulated the osteoblast proliferation, promoting better osteointegration [33,34,35].

A remarkable number of regenerating muscle fibres emerged in close proximity to the implant during the postoperative month. Such a phenomenon proved the healing process to be in progress, as the granulation tissues were being gradually replaced by the regenerating muscle fibres. Moreover, the presence of a small amount of the granulation tissue and the regenerating fibres adhering to the prosthesis confirmed not only the better muscle reconstruction but also more promising eventual osteointegration. The observed high OCC enzyme activity in the tissue directly around the implant proved the lack of biomaterial cytotoxicity. The regenerating muscle tissue was characterized by the proper morphology and the mature muscles displayed the desired mosaic arrangement.

During the experiment a small capsule of the connective tissue was revealed close to the implant, resulting from the fast proliferation of fibroblasts. It was an adverse phenomenon, possibly leading to the implant encapsulation and constriction. It is worth noting that the connective tissue overgrowth influences the proper bone/implant fixation; thus, it should be limited or eliminated in the middle ear implantations where the surrounding environment are bone structures [36,37]. Although the tested prostheses are supposed to reconstruct the bones, it was purposeful to conduct the experiments on the muscles, as soft tissues reveal more severe immunological reaction to a foreign body [38].

### 3.4. Micro-CT Observations

**Figure 9:**
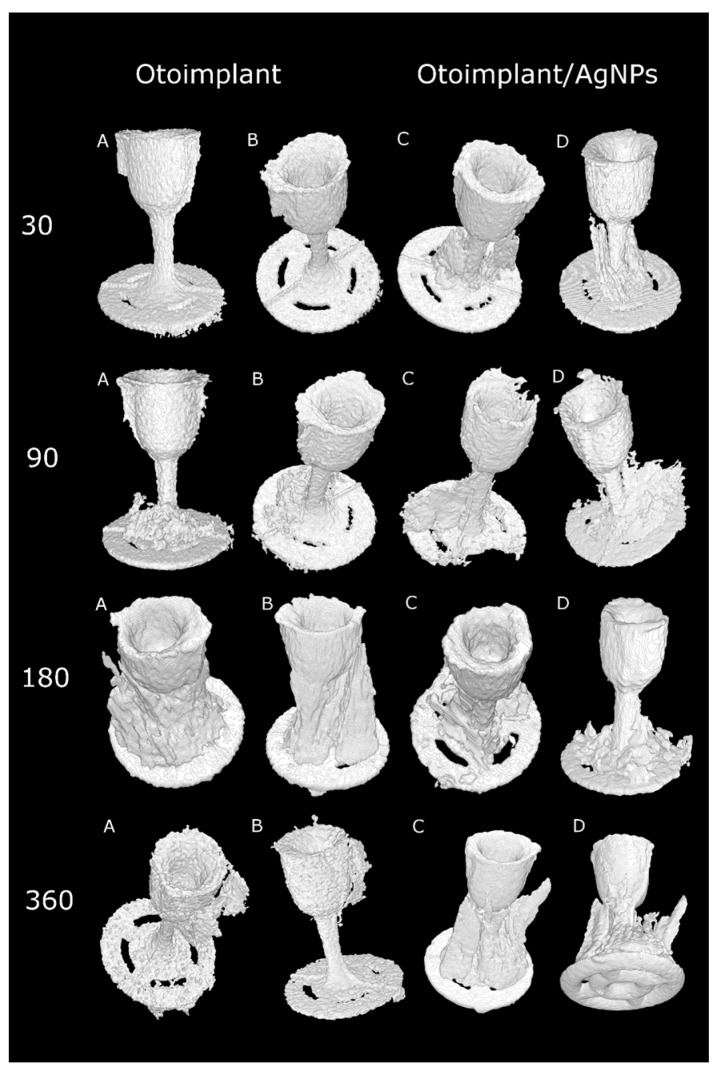
Volume reconstructions showing otoimplant (**A**,**B**—different orientation of prosthesis) and otoimplant/AgNPs (**C**,**D**—different orientation of prosthesis) after 30, 90, 180 and 360 days of the implantation in rats muscles, surrounding granulation tissue visible.

The micro-CT results confirmed that the muscle tissue regeneration was faster for the implants modified with silver nanoparticles (Figure 9). It was particularly evident in the course of time, after longer implantation periods. Thirty days after the operation there was definitely more granulation tissue around the Ag-doped implant than around the pure polymer implant. In both types of prostheses, the muscles were damaged during the surgery so they did not resemble the proper mosaic. However, after 90 days the correct reconstruction of the muscles was noticeable for both types of implants. The micro-CT after 30 days revealed the bigger granulation area of the otoimplant/AgNPs samples, which confirmed the results of histochemical observations obtained at the same time (Figure 8). Three months after the operation it was observed that the area of granulation tissue was decreasing rapidly for the silver-modified implant. In the case of the non-modified implant, the area of granulation tissue was also higher in comparison to the results taken after 30 days. After 180 days, the micro-CT reconstruction revealed that tissue rebuilding was less evident for the otoimplant/AgNPs. However, having analyzed histochemical results along with the Image J evaluation and the micro-CT reconstruction, it may be assumed that after 360 days of the implantation the tissue area around AgNPs enriched implant was comparable to the one after 180 days. The micro-CT showed granulation tissue together with other tissues, therefore, it was necessary to compare the micro-CT results to the histochemical reactions. The CT results were less specific than the histochemical tests. The micro-computed tomography offered more comprehensive and accurate information than traditional methods [39,40]. The 3D visualization based on micro-CT allowed us to observe the implant behaviour in the tissue via the ex vivo imaging. Therefore, the ex vivo observations clearly showed how the muscles surrounding the implants were regenerating with time and both the composition of the implant material and the prosthesis shape facilitated the muscle tissue regeneration. Silver nanoparticles accelerated the healing process. Both the micro-CT imaging and microscopic observations confirmed that the regeneration around the cup was much faster than around the antenna whose complex structure hindered the process. The natural reaction of the muscles to the foreign object also made the implant unstable. The prosthesis of an unusual shape and certain density and stiffness irritated the surrounding tissue.

The histochemical tests and micro-CT proved the AgNPs-modified implant to be better integrated with the regenerated muscle tissue than the pure ABS prosthesis. The images of otoimplant/AgNPs in subsequent time intervals showed that tissue around the implant was growing with the passage of time.

### 3.5. Silver Ions Release by Modified Implants

**Figure 10:**
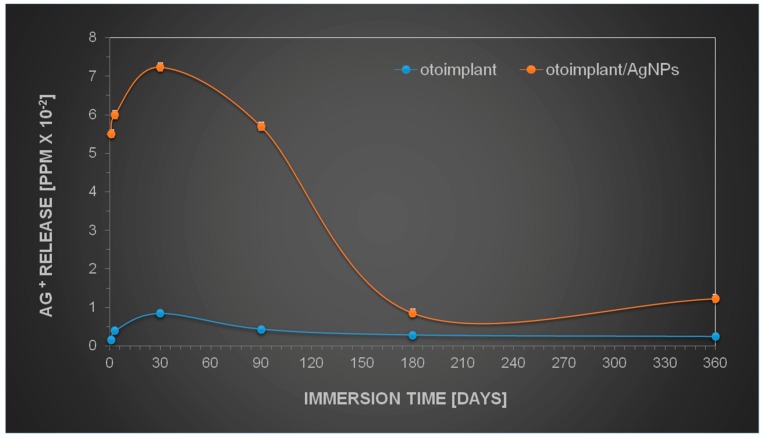
Kinetics of silver ions release during one-year incubation in UHQ water.

The results obtained by the observation of implant tissue samples (Figure 6, Figure 7, Figure 8 and Figure 9) were then compared to the release of silver from AgNPs-implants over one year of incubation in water. Figure 10 shows that the Ag^+^ release depended on the immersion time, increasing as a function of time. However, the highest increase was observed during the first month of incubation. The gradual silver ions decrease was observed from the 3rd month on, whereas between the 6th and 12th month the release was only marginal.

The similar behaviour was expected in the in vivo studies. The gradual release of silver seemed to be an advantageous phenomenon, since the Ag-modified implant was surrounded by the juvenile muscle tissue without the separating granulation layer. The research also proved that the amount of silver released into the tissue was safe and probably advantageous for the faster muscle regeneration. The literature has reported silver nanoparticles to be nontoxic to humans and very effective against bacteria, viruses, and other eukaryotic micro-organisms at very low concentrations and without side effects. Jeong S.H. et.al. [41] proved that for the silver nanoparticles in the content of 0.1% materials exhibit excellent antibacterial effect (bacterial reduction of 99.9%), but for the micron-sized silver in the content above > 0.5 the antibacterial activity was determined as good. A variety of dressings that contain and release silver ions at the wound surface provide controlled release of ions through a slow but sustained release mechanism, which prevents toxicity yet ensures delivery of a therapeutic dose of silver ions to the wound [42].

There was no strong polymer—filler interactions between AgNPs and the polymer matrix in the nanocomposite networks. This phenomenon loosened the molecular packing of polymeric chains near the nanoparticles and caused an increase in free volume in the nanocomposite networks [43]. The aqueous medium easily diffused into the empty areas and led to the Ag oxidation. Therefore, the water diffusion in the composite sample was expected to result in the higher Ag^+^ release [44]. The long-term release seemed to be an important factor in regard to practical applications. The subsequent release of silver ions occurred in the interior part of the specimen where water had to cross the diffusion barrier, which inhibited the oxidation process. Conversely, in some cases, polymers could be porous enough to allow water to pass through the polymer and, subsequently, the silver nanoparticles could diffuse out of the polymer [45]. Such a mechanism could be responsible for the prolonged antibacterial efficiency. On the other hand, Helttunen et al. [46] as well as McShan et al. [47] proved that the extensive release of silver from the Ag^+^ or AgNPs-doped materials was environmentally hazardous and toxic to humans. On the contrary, our long-time observations revealed no toxicity. The research proved that using nanoparticles as active components in composite materials instead of conventional chemical products, e.g., ethanol or bleach, provided the long-lasting bactericidal efficiency with no toxic effect [48]. Therefore, the assumed level of silver concentration in our implant revealed antibacterial efficacy, yet it was not toxic to animals and humans.

### 3.6. Physicochemical Properties of Prostheses

**Figure 11:**
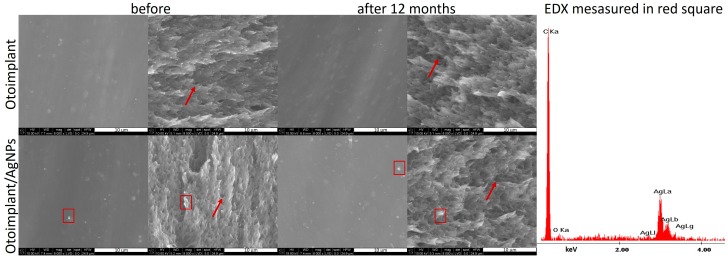
SEM images showing the otoimplant and otoimplant/AgNPs before and after 12 months incubation, surface and cross-section were collected alternately from left to right. On the right—the EDX spectrum collected in the point of the thick red square indicated the presence of AgNPs.

The scanning electron microscopy observation showed that the surface of both otoimplant and otoimplant/AgNPs was smooth (Figure 11). Silver nanoparticles observed as a light area on SEM images were homogenously distributed in the polymer matrix. The double-cycle injection moulding and extrusion were applied to limit the aggregation of silver nanoparticles. Therefore, the size of nanoparticles remained the same after their integration into polymer. However, even such a precise technology could lead to the prevalence of small aggregates (red square on SEM images proved by EDX spectrum). The observations performed after one year of incubating the samples in deionized water (36 °C) revealed no changes on the surface and in the cross-section. SEM images of the cross-section of polymeric and composite implants showed some porous microstructure (marked with arrows), which could facilitate the silver ions release.

**Figure 12:**
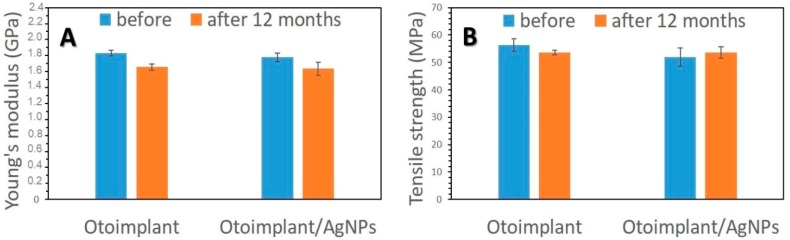
Young’s modulus (**A**) and tensile strength (**B**) of otoimplant and otoimplant/AgNPs before and after 12-month incubation in deionized water.

As it was proved in our previous work [28], the long-term bactericidal effect was sustained by the gradual release of silver due to the efficient dispersion of its nanoparticles in the polymer matrix. On the other hand, the surfaces of both the pure implant and implant modified with AgNPs were smooth, which inhibited the bacteria colonization. It is well-known from the literature that the increased roughness facilitates adhesion of osteoblasts to the material surface [49,50]. Higher roughness parameters promote adhesion of bacteria, microbial proliferation and formation of biofilms, which may lead to inflammatory processes, cell necrosis and even rejection of the implanted material [51]. Our assumption was to minimize the bacteria colonization thanks to a remarkably smooth surface. Even if bacteria attached to the porous surface, the gradual silver ions release would guarantee antibacterial efficacy. The conducted mechanical tests proved that the force-elongation curves were similar for both groups of materials—the pure polymers and the composites. Such results suggested that the proposed technology of obtaining polymeric paddles by means of extrusion and injection moulding did not impoverish the mechanical properties of the tested materials. Young’s modulus and tensile strength (Figure 12) remained at the same level both for the samples before and after the incubation in deionized water. The addition of 0.1 wt. % of AgNPs did not change tensile strength but slightly decreased Young’s modulus of the composite samples. Therefore, AgNPs had no negative effects on the mechanical properties of ABS. In addition, low concentrations of nanoparticles eliminated agglomeration adversely affecting the material’s properties. Moreover, the mechanical properties of composites depended on the amount and size of incorporated nanoparticles. Namely, smaller nanoparticles were more effective against bacteria [52]. However, when the AgNPs were smaller than 3 nm, they were more cytotoxic than larger particles—25 nm [15]. On the other hand, the particles measuring 50 nm or more increased the flexural strength of the composite containing 0.2 wt. % of AgNPs. The 40 nm particles did not affect the mechanical parameters of these composites [53]. The findings proved that the mechanical properties of the otoimplant did not change after the incorporation of AgNPs even after the 12-month incubation. Such results suggested that our prosthesis would be stable in clinical performance.

## 4. Conclusions

Nanotechnology offers great opportunities to improve properties of medical devices. Our in vivo research has proven that the prosthesis made of ABS enriched with silver nanoparticles is biocompatible with the surrounding tissue. Moreover, the AgNPs incorporated into the polymer medical devices ensure a long-lasting antibacterial effect combined with the lack of inflammation or toxic reaction. The addition of silver nanoparticles accelerates the healing process, which is crucial to the length of convalescence after the ossicle chain reconstruction. The microstructural observations as well as mechanical tests have proven the biostability of both prostheses. The low concentration addition of AgNPs to the polymer matrix does not alter the material’s mechanical and microstructure properties. The obtained results are the promising basis for further research on the implant prototype that may become an alternative to the devices already available on the medical market.
